# Target product profile: aerosolized surfactant for neonatal respiratory distress

**DOI:** 10.2471/BLT.23.289727

**Published:** 2023-03-02

**Authors:** Suman Rao, Karen Edmond, Rajiv Bahl

**Affiliations:** a Department of Maternal, Newborn, Child and Adolescent Health and Ageing, World Health Organization, Avenue Appia 20, 1211 Geneva 27, Switzerland.

## Abstract

Treatment with surfactant has been found to improve the survival rate of neonates with respiratory distress syndrome, particularly preterm infants. However, surfactant is usually administered by endotracheal intubation and generally only in level-3 neonatal intensive care units. Recent improvements in aerosolization technology have raised the possibility that aerosolized surfactant could now be given in wider range of settings, including resource-poor settings. Consequently, the World Health Organization has developed a target product profile for product developers that describes the optimal and minimal characteristics of an aerosolized surfactant for treating neonates with respiratory distress syndrome in low- and middle-income countries. Development of the target product profile involved a scoping review of systematic reviews and target product profiles of aerosolized surfactant, the constitution of an international expert advisory group, consultations with medical professionals from a wide range of countries and a public consultation. The resulting target product profile specifies that the surfactant and its associated aerosolization device should ideally, among other characteristics: (i) be at least as safe and effective as current intratracheal surfactant; (ii) produce a rapid clinical improvement; (iii) be easy to transport and use (e.g. by nurses in level-2 health-care facilities in low- and middle-income countries); (iv) be affordable for low- and middle-income countries; and (v) be stable when stored in hot and humid conditions. In addition, the aerosolization device should be capable of daily use for many years. The introduction of an effective aerosolized surfactant globally could substantially reduce neonatal mortality due to respiratory distress syndrome.

## Résumé

Le traitement par surfactant permet d'améliorer le taux de survie des nouveau-nés souffrant d'un syndrome de détresse respiratoire, en particulier les nourrissons prématurés. Pourtant, ce surfactant est généralement administré par intubation endotrachéale et, la plupart du temps, uniquement dans les unités de soins intensifs néonatals de niveau 3. Les récents progrès réalisés dans le domaine des technologies d'aérosolisation laissent entrevoir la possibilité d'administrer dorénavant un surfactant en aérosol dans d'autres cadres, y compris dans des lieux où les ressources sont limitées. L'Organisation mondiale de la Santé a donc développé un profil de produit cible à l'attention des laboratoires, qui détaille les caractéristiques minimales et optimales d'un surfactant en aérosol destiné à la prise en charge des nouveau-nés souffrant d'un syndrome de détresse respiratoire dans les pays à revenu faible et intermédiaire. Ce document est le fruit d'une analyse exploratoire de revues systématiques et de profils de surfactants en aérosol; un groupe consultatif d'experts internationaux a été constitué, des professionnels de la santé originaires de nombreux pays ont été sollicités, et une consultation publique a été organisée. Le profil de produit cible qui en résulte précise que le surfactant et son dispositif d'aérosolisation doivent idéalement présenter une série de caractéristiques, notamment: (i) être au moins aussi sûrs et efficaces que le surfactant intratrachéal actuel; (ii) entraîner une amélioration clinique rapide; (iii) être faciles à transporter et à utiliser (par exemple par le personnel infirmier de niveau 2, au sein d'établissements de santé dans des pays à revenu faible et intermédiaire); (iv) être abordables pour les pays à revenu faible et intermédiaire; et enfin, (v) demeurer stables quand ils sont stockés dans un environnement chaud et humide. En outre, le dispositif d'aérosolisation doit pouvoir être employé au quotidien pendant plusieurs années. Le lancement d'un surfactant en aérosol à l'échelle mondiale pourrait réduire considérablement la mortalité néonatale due au syndrome de détresse respiratoire.

## Resumen

Se ha observado que el tratamiento con sustancias tensioactivas mejora la tasa de supervivencia de los neonatos con síndrome de dificultad respiratoria, especialmente los prematuros. Sin embargo, la sustancia tensioactiva suele administrarse mediante intubación endotraqueal y, por lo general, solo en unidades de cuidados intensivos neonatales de nivel 3. Las mejoras recientes en la tecnología de aerosolización han planteado la posibilidad de que la sustancia tensioactiva en aerosol se pueda administrar ahora en más entornos, incluidos los de escasos recursos. En consecuencia, la Organización Mundial de la Salud ha desarrollado un perfil de producto específico para los desarrolladores de productos que describe las características óptimas y mínimas de una sustancia tensioactiva en aerosol para el tratamiento de neonatos con síndrome de dificultad respiratoria en países de ingresos bajos y medios. El desarrollo del perfil de producto específico implicó una revisión del alcance de las revisiones sistemáticas y los perfiles de producto específicos de la sustancia tensioactiva en aerosol, la constitución de un grupo asesor internacional de expertos, consultas con profesionales médicos de diversos países y una consulta pública. El perfil de producto específico obtenido indica que lo ideal sería que la sustancia tensioactiva y su dispositivo de aerosolización asociado tuvieran, entre otras, las siguientes características: (i) ser al menos tan seguros y eficaces como la sustancia tensioactiva intratraqueal actual; (ii) producir una mejora clínica rápida; (iii) ser fáciles de transportar y utilizar (p. ej. por el personal de enfermería de los centros sanitarios de nivel 2 de los países de ingresos bajos y medios); (iv) ser asequibles para los países de ingresos bajos y medios; y (v) ser estables cuando se almacenan en condiciones de calor y humedad. Además, el dispositivo de aerosolización se debería poder utilizar a diario durante muchos años. La introducción de una sustancia tensioactiva en aerosol eficaz a nivel mundial podría reducir de manera sustancial la mortalidad neonatal causada por el síndrome de dificultad respiratoria.

## موجز

لقد تبين أن العلاج بالمادة الفعالة بالسطح الرئوي يؤدي إلى تحسين معدل البقاء على قيد الحياة لدى حديثي الولادة المصابين بمتلازمة عسر التنفس، وخاصةً الأطفال المبتسرين. إلا أنه عادةً ما يتم إعطاء المادة الفعالة بالسطح الرئوي عن طريق إدخال أنبوب داخل الرغام، ويتم ذلك عمومًا في وحدات العناية المركزة لحديثي الولادة من المستوى 3 فقط. لقد أدت التحسينات الأخيرة في تقنية التحويل إلى رذاذ إلى زيادة إمكانية إعطاء المادة الفعالة بالسطح الرئوي عل شكل رذاذ الآن في مجموعة أكبر من الأماكن، بما في ذلك الأماكن محدودة الموارد. وبناءً على ذلك، وضعت منظمة الصحة العالمية ملف تعريف للمنتج المستهدف لصالح مطوري المنتجات يصف السمات المثالية والدنيا للمادة الفعالة بالسطح الرئوي على شكل رذاذ لمعالجة حديثي الولادة المصابين بمتلازمة عسر التنفس في البلدان ذات الدخل المنخفض والدخل المتوسط. تضمّن وضع ملف تعريف المنتج المستهدف مراجعة لتحديد نطاق المراجعات النظامية وملفات تعريف المنتجات المستهدفة للمادة الفعالة بالسطح الرئوي على شكل رذاذ، وقواعد المجموعة الاستشارية للخبراء الدوليين، والمراجعات مع الأخصائيين الطبيين من مجموعة واسعة من البلدان والمشاورة العامة. يحدد ملف تعريف المنتج المستهدف الناتج أن المادة الفعالة بالسطح الرئوي وجهاز التحويل إلى رذاذ المرتبط به ينبغي أن يتسم بما يلي إلى جانب سمات أخرى: (1) أن يكون آمنًا وفعالاً بما لا يقل عن المادة الفعالة بالسطح الرئوي الرغامي الحالي، و(2) أن يؤدي إلى تحسن سريري سريع، و(3) أن يكون سهلاً في النقل والاستخدام (بواسطة الممرضين مثلاً في منشآت الرعاية الصحية من المستوى 2 في البلدان منخفضة الدخل ومتوسطة الدخل)، و(4) أن يكون معقول التكلفة بالنسبة إلى البلدان منخفضة الدخل ومتوسطة الدخل، و(5) أن يكون بحالة مستقرة عند تخزينه بظروف حارة ورطبة. كما ينبغي أن يكون جهاز التحويل إلى رذاذ صالحًا للاستخدام اليومي على مدار العديد من السنوات. إن تقديم مادة فعالة بالسطح الرئوي فعالة عالميًا على شكل رذاذ، يمكن أن يقلل بشدة من معدل وفيات حديثي الولادة بسبب متلازمة عسر التنفس.

## 摘要

表面活性剂疗法已被发现可提高患有呼吸窘迫综合征的新生儿（特别是早产儿）的存活率。然而，表面活性剂通常仅在三级新生儿重症监护病房通过气管插管方式给药。近期雾化技术的改进提高了雾化肺表面活性剂在更广泛的环境（包括资源贫乏的环境）中使用的可能性。因此，世界卫生组织为产品开发人员制定了目标产品简介，描述了低收入和中等收入国家用于治疗新生儿呼吸窘迫综合征的雾化肺表面活性剂的最佳和最基本特征。目标产品简介的制定过程包括对系统评价和雾化肺表面活性剂目标产品简介进行概况性综述，组成国际专家咨询小组，与来自多个国家的医疗专业人员进行磋商，以及进行公众咨询。最终的目标产品简介规定表面活性剂及其相关的雾化器在理想状态下应具有以下特征：(i) 至少与目前气管给药的表面活性剂一样安全有效；(ii) 能够迅速改善临床症状；(iii) 便于运输和使用（例如，在低收入和中等收入国家可供二级医疗卫生机构的护士使用）；(iv) 低收入和中等收入国家能够负担得起；以及 (v) 在湿热条件下储存时能够保持稳定性。此外，雾化器应能够在正常日常使用的情况下使用多年。在全球范围内引入一种有效的雾化肺表面活性剂可以在很大程度上降低由呼吸窘迫综合征引起的新生儿死亡率。

## Резюме

Установлено, что лечение сурфактантом повышает коэффициент выживаемости новорожденных с респираторным дистресс-синдромом, особенно недоношенных детей. Однако сурфактант обычно вводят путем эндотрахеальной интубации и, как правило, только в отделениях интенсивной терапии новорожденных третьего уровня. Недавние усовершенствования в технологии производства аэрозолей привели к тому, что теперь аэрозольное введение сурфактанта может осуществляться в более широком диапазоне условий, включая условия с ограниченными ресурсами. Поэтому Всемирная организация здравоохранения разработала целевой профиль препарата для разработчиков, в котором описаны оптимальные и минимальные характеристики сурфактанта в виде аэрозоля для лечения новорожденных с респираторным дистресс-синдромом в странах с низким и средним уровнем доходов. Для разработки целевого профиля препарата был проведен обзор систематических обзоров и целевых профилей препаратов сурфактанта в виде аэрозоля, создана международная экспертная консультативная группа, проведены консультации с медицинскими работниками из широкого круга стран и медицинские консультации для населения. Полученный целевой профиль препарата указывает, что сурфактант и связанное с ним устройство для аэрозольного введения должны обладать оптимальными среди прочих характеристиками: (i) быть как минимум таким же безопасным и эффективным, как существующий сурфактант для эндотрахеального введения; (ii) вызывать быстрое клиническое улучшение; (iii) быть простым в транспортировке и использовании (например, медсестрами в медицинских учреждениях второго уровня в странах с низким и средним уровнем доходов); (iv) быть доступным по цене для стран с низким и средним уровнем доходов; (v) сохранять стабильность при хранении в условиях жаркого и влажного климата. Кроме того, устройство для аэрозольного введения должно быть пригодным для ежедневного использования в течение многих лет. Использование эффективного сурфактанта в виде аэрозоля в глобальном масштабе может существенно снизить смертность новорожденных от респираторного дистресс-синдрома.

## Introduction

Respiratory distress syndrome causes approximately 15% of all neonatal deaths globally.^1^^,^^2^ The administration of antenatal corticosteroids and early initiation of continuous positive airway pressure both have a substantial impact on mortality from respiratory distress.^3^^–^^5^ In addition, there is clear evidence that treatment with surfactant, which decreases surface tension at the air–liquid interface in the lungs, reduces the severity of respiratory distress syndrome in preterm infants and improves survival.^6^^–^^8^ Accordingly, surfactant is used routinely in high-income countries to treat preterm infants. Currently, however, surfactant administration usually requires endotracheal intubation to enable direct instillation of liquid surfactant into the trachea. Then the infant often requires mechanical ventilation until respiratory status improves.

The World Health Organization (WHO) recommends the use of animal-derived or protein-containing synthetic surfactant for preterm neonates with respiratory distress syndrome who are intubated and undergoing mechanical ventilation.^9^^,^^10^ However, current WHO guidelines state that surfactant should be given only in health-care facilities where blood gas analysis and newborn nursing care and monitoring are available.^9^ Additionally, the cost of current formulations remains high and poses a significant barrier to access. As a result, surfactant use in low- and middle-income countries is challenging.

To date, surfactant has generally been used only in level-3 tertiary neonatal intensive care units and has been out of reach for many level-2 special care baby units in district-level facilities.^11^ There is a need for a low-cost surfactant that can be administered without intubation and mechanical ventilation, and is as efficacious and safe as standard surfactant. Many different surfactants and administration methods have been trialled, including less-invasive surfactant administration, which involves administering surfactant using a small catheter.^12^^,^^13^ Surfactant delivery via laryngeal mask airway or pharyngeal instillation are other commonly used methods, but they also involve passing tubes into the oro- or naso-pharynx.^14^

Nebulized surfactant has been investigated for many years.^12^ Older jet and ultrasonic nebulizers have several limitations, such as denaturation of surfactant and surfactant being trapped in tubing and filters.^15^ Recent improvements in aerosolization technology have made it possible to produce small particles that enable a higher aerosolized dose to be deposited in the lungs.^12^^,^^15^ Aerosolized surfactant formulations will probably cost less and be easier to administer than conventional formulations. As a result, they could be used in district level-2 special care baby units in every country around the world. However, aerosolized surfactant is relatively new, and currently it is unclear what the best formulation or form of administration would be for low-resource settings, or whether storage and treatment are feasible.

WHO has now developed a target product profile that describes the optimal and minimal characteristics of an aerosolized surfactant that can be used for neonates (i.e. infants younger than 28 days) with respiratory distress syndrome in low- and middle-income countries. The profile’s purpose is to inform product developers about the key performance specifications and test characteristics of an aerosolized surfactant that will meet the needs of end-users in these countries. The overarching principles underlying the target product profile are that the aerosolized surfactant and its delivery system should: (i) be as safe and efficacious as conventional surfactant formulations; (ii) be capable of being administered and monitored in low- and middle-income countries; (iii) be affordable for these countries; and (iv) be capable of being used in special care baby units in level-2 or higher facilities in all countries globally.

## Methods

A scoping review of systematic reviews and target product profiles of aerosolized surfactant for preterm neonates was performed. Then WHO developed a preliminary outline of a target product profile that focused on the needs of low- and middle-income countries. In this process, the principles outlined by WHO’s Science Division in 2022 for developing target product profiles were followed.^16^

Subsequently, an expert advisory group of 10 external experts from several WHO regions was identified by WHO’s secretariat by scanning the literature and contacting networks and colleagues. Group members were chosen to ensure technical expertise, interest, wide geographical representation and a gender balance. The result was a diverse group of individuals with expertise in neonatology, clinical practice, health promotion, policy and programmes, administration and service delivery (Table 1).

**Table 1:** Expert advisory group, development of a target product profile for aerosolized surfactant therapy for neonates in low- and middle-income countries, 2022

Name	Affiliation	Country of residence
Gul Ambreen	Department of Pharmacy, Aga Khan University Hospital, Karachi, Pakistan	Pakistan
Shabina Ariff	Department of Paediatrics and Child Health, Aga Khan University, Karachi, Pakistan	Pakistan
Tasmin Bota	Parent representative, Johannesburg, South Africa	South Africa
Queen Dube	Ministry of Health, Lilongwe, Malawi	Malawi
Meena Joshi	Nurse educator, All India Institute of Medical Science, New Delhi, India	India
Kondwani Kawaza	Kamuzu University of Health Sciences, Blantyre, Malawi	Malawi
Siddarth Ramji	Retired consultant neonatologist, Maulana Azad Medical College, New Delhi, India	India
Muhammad Shariful Islam	Assistant director and programme manager, National Newborn Health Program (NNHP) and Integrated Management of Childhood Illness (IMCI), Directorate General of Health Services, Dhaka, Bangladesh	Bangladesh
Hoang Tran	Da Nang Hospital for Women and Children, Da Nang, Viet Nam	Viet Nam
Bogale Worku	Ethiopian Pediatric Society, Addis Ababa, Ethiopia	Ethiopia

On 11 and 12 January 2022, a consultative meeting was held for individuals involved in the use of surfactant, such as doctors, nurses, pharmacists, administrators, procurement officers, programme managers, policy-makers and members of academic organizations. There were 37 attendees from 16 countries, including 13 low- and middle-income countries. All members of the expert advisory group and participants in the consultative meeting completed WHO declaration of interest and confidentiality forms. No individual had a conflict of interest. In addition, the biographies of expert advisory group members were posted on WHO’s website at the time of the consultative meeting.

The preliminary target product profile was developed further on the basis of the consultative meeting and an initial, or zero, draft was produced. The zero draft was posted on WHO’s website on 20 June 2022 for public consultation with the purpose of inviting comments and suggestions. Subsequently, 22 sets of comments were received and incorporated into a revised version of the target product profile, which was reviewed and finalized by the expert advisory group and WHO’s secretariat. The final target product profile is now available online.^17^

## Target product profile

### Use, efficacy and safety of aerosolized surfactant

According to the target product profile,^17^ aerosolized surfactant should preferably be suitable for treating respiratory distress syndrome in all neonates, regardless of the use of non-invasive respiratory support devices (e.g. continuous positive airway pressure). The minimum requirement is that it should be suitable for use in neonates: (i) who are not responding to non-invasive respiratory support; or (ii) who have a birth weight of 1000 g or more or a gestational age of 28 weeks or more. The surfactant should preferably be suitable for use in special care baby units in level-2 health-care facilities in low- and middle-income countries, regardless of their referral network.^11^ At a minimum, it should be suitable for use in facilities able to refer neonates to level-3 facilities. Preferably, users would include nurses, all medical doctors, paediatricians and their equivalents. The minimum user group would comprise medical doctors with some training in neonatal care, paediatricians and their equivalents.

With regard to efficacy, aerosolized surfactant use should preferably result in a 30% or greater reduction in mortality or in failure to respond to continuous positive airway pressure among infants who receive continuous positive airway pressure plus surfactant, compared to infants who receive continuous positive airway pressure alone.^18^ The minimum efficacy requirement is 20%. Moreover, efficacy should be equal to, or better than, that currently achieved by intratracheal surfactant. Preferably, the aerosolized surfactant should result in fewer adverse events than current surfactant therapy. At a minimum, the adverse event rate should be comparable to that with current surfactant.

### Drug dosing and administration

The aerosolized surfactant should preferably need only one dose for maximum effect, which could be administered up to 48 to 72 hours after birth. Moreover, the maximum effect should be at least equivalent to that with current surfactant preparations. The minimum requirement is that three doses or fewer would be needed for maximum effect, and that administration could be started up to 12 hours after birth. Ideally, the dose administered should be independent of body weight or dosing should be determined by body weight band (e.g. less than 0.5 kg, 0.6 kg to 1.0 kg, 1.1 kg to 1.5 kg and so on). The minimum requirement is that dosing should be determined by body weight in mL/kg.

During administration of the aerosolized surfactant, there should preferably be no interruption to the operation of any non-invasive respiratory support device. The minimum requirement is that the device should be interrupted for less than 5 minutes. A measurable clinical improvement (e.g. improved oxygenation, reduced oxygen requirement or less breathing effort) should preferably be apparent within 15 minutes of treatment completion. The minimum requirement is an improvement within 60 minutes. In addition, the maximum benefit of treatment should preferably be achieved within 2 hours of treatment completion. The minimum requirement is within 6 hours. Preferably, at least 30% of the surfactant should be deposited in the alveoli (i.e. in the distal lung region and parenchyma). The minimum requirement is 15%.

### Drug formulation and packaging

The shelf-life stability of the aerosolized surfactant should preferably be at least 36 months in hot and humid conditions (defined as a temperature of 30°C or more and a relative humidity of 75%) and the in-use stability should be at least 120 minutes under these conditions. The minimum requirements are a shelf-life stability of at least 24 months at 2°C to 8°C and an in-use stability of at least 60 minutes in hot and humid conditions.

The aerosolized surfactant should preferably be synthetic. The minimum requirement is an animal-derived surfactant or protein-containing synthetic surfactant. The drug and its associated aerosolization device must be packaged separately, and the final packaging should preferably be capable of being shipped by road, rail, air or sea without the need for a cold chain. At a minimum, the aerosolization device should be capable of being shipped by road, rail, air or sea without the need for a cold chain, and the drug should be capable of being shipped through a cold chain. The drug and device must be capable of being disposed of via established pharmaceutical waste streams.

### Aerosolization device

The aerosolization device must be developed in accordance with appropriate regulatory guidance and standards. Preferably, it should work on both battery and external electricity power sources. At a minimum, it could be plugged into an external power source. The device must be capable of being used by both right- and left-handed people, and must be compact and easy to use and transport.

Ideally, it should be possible to learn the skills required to operate the aerosolization device within 2 to 3 hours of training. The minimum requirement is that skills should be learned within 2 to 3 days. It should be possible to set up the device in fewer than five steps and within 5 minutes or less. The minimum requirement is that it should take 10 steps and 10 minutes to set up.

The aerosolization device must be designed to ensure that only one unit dose can be administered at a time, and it must be capable of being used for several patients with the aid of an appropriate cleaning and disinfecting regimen. In addition, it must meet the standards for non-invasive respiratory support devices and nebulization devices, and should preferably function for at least 5 years of daily use. At a minimum, it should function for 2 years of daily use.

### Cost

The cost of the aerosolized surfactant should preferably be less than 5 United States dollars (US$) per dose delivered, the aerosolization device should cost less than US$100 and the total cost per infant should be less than US$25. The minimum requirements are that the aerosolized surfactant should cost less than US$50 per dose, the device should cost less than US$1000 and the total cost per infant should be less than US$220.

## Conclusion

Working with an international expert advisory group and after consultations with medical professionals from a wide range of countries and a public consultation, WHO developed a target product profile for an aerosolized surfactant for use in neonates with respiratory distress syndrome in low- and middle-income countries. According to the product profile, the surfactant and its associated aerosolization device should ideally: (i) be affordable; (ii) be stable when stored in hot and humid conditions; (iii) be easy to transport and use (e.g. by nurses in level-2 health-care facilities in low- and middle-income countries); (iv) produce a rapid clinical improvement; and (v) be as, or more, effective than current intratracheal surfactant. In addition, the aerosolization device should last for many years of daily use. The introduction of an effective aerosolized surfactant globally would substantially reduce neonatal mortality due to respiratory distress syndrome.
